# Structure of an anti-PEG antibody reveals an open ring that captures highly flexible PEG polymers

**DOI:** 10.1038/s42004-020-00369-y

**Published:** 2020-09-08

**Authors:** Justin T. Huckaby, Tim M. Jacobs, Zhongbo Li, Robert J. Perna, Anting Wang, Nathan I. Nicely, Samuel K. Lai

**Affiliations:** 1 UNC/NCSU Joint Department of Biomedical Engineering, School of Medicine, University of North Carolina-Chapel Hill, Chapel Hill, NC 27599 USA; 2 Division of Pharmacoengineering and Molecular Pharmaceutics, Eshelman School of Pharmacy, University of North Carolina-Chapel Hill, Chapel Hill, NC 27599 USA; 3 Department of Psychology and Neuroscience, College of Arts and Sciences, University of North Carolina-Chapel Hill, Chapel Hill, NC 27599 USA; 4 Department of Pharmacology, School of Medicine, University of North Carolina-Chapel Hill, Chapel Hill, NC 27599 USA; 5 Department of Microbiology and Immunology, School of Medicine, University of North Carolina-Chapel Hill, Chapel Hill, NC 27599 USA

**Keywords:** X-ray crystallography, Immunochemistry, Proteins, Biomaterials - proteins, Drug delivery

## Abstract

Polyethylene glycol (PEG) is a polymer routinely used to modify biologics and nanoparticles to prolong blood circulation and reduce immunogenicity of the underlying therapeutic. However, several PEGylated therapeutics induce the development of anti-PEG antibodies (APA), leading to reduced efficacy and increased adverse events. Given the highly flexible structure of PEG, how APA specifically bind PEG remains poorly understood. Here, we report a crystal structure illustrating the structural properties and conformation of the APA 6-3 Fab bound to the backbone of PEG. The structure reveals an open ring-like sub-structure in the Fab paratope, whereby PEG backbone is captured and then stabilized via Van der Waals interactions along the interior and exterior of the ring paratope surface. Our finding illustrates a strategy by which antibodies can bind highly flexible repeated structures that lack fixed conformations, such as polymers. This also substantially advances our understanding of the humoral immune response generated against PEG.

## Abstract (web summary)

Polyethylene glycol (PEG) is a widely used, biocompatible material, but can promote the development of anti-PEG antibodies. Here, crystal structures of an anti-PEG antibody binding fragment bound to PEG are reported and key binding residues are identified by site-directed mutagenesis.

## Introduction

Polyethylene glycol (PEG) is a simple yet highly versatile synthetic polymer with the chemical structure (CH_2_CH_2_O)_*n*_. By nature of this repeat ethylene glycol structure, PEG is both exceedingly hydrophilic and highly flexible. PEG chains create a stable hydration layer through hydrogen bonding with nearby water molecules^1,2^. These physicochemical properties increase drug solubility and stability^3^, reduce renal filtration^4^, and enable PEGylated surfaces to effectively resist nonspecific protein adsorption^4,5^, including by opsonins that drive rapid clearance from the systemic circulation. These highly favorable “stealth” properties have led to wide adoption of PEG as a quintessential component in many drug delivery systems and protein therapeutics over the past few decades^6^.

Nearly 20 PEGylated systems have been approved by the United States Food and Drug Administration (US FDA), and more than a dozen others are currently in clinical trials^7^. The vast majority of these PEGylated therapeutics have proven to be safe and efficacious over repeated dosing. For example, PEGylated recombinant human coagulation factor VIII proteins, including Adynovate^®^ and Esperoct^®^, are used chronically as replacement therapy to treat hemophilia without evidence of any PEG-related safety concerns^8^. PEGylated biotherapeutics utilizing very high MW PEG, such as MIRCERA (30 kDa PEG) and CIMZIA (40 kDa branched PEG), have also shown no safety or efficacy issues related to their conjugated PEG moieties in over a decade^9^.

Unfortunately, for a small number of PEGylated therapeutics, there is now firm evidence that anti-PEG antibodies (APA) can be induced in patients. Indeed, APA is directly responsible for the loss of efficacy in nearly half of the patients receiving either PEG-asparaginase and PEG-uricase^10,11^, as well as the early termination of a number of clinical trials due to APA-triggered adverse events^12–14^. This came as a surprise to many in the field; due to PEG’s highly flexible nature and ability to resist protein adsorption, PEG was long assumed by most to be non-immunogenic^15,16^. The first evidence that antibodies can be induced against PEG can be traced back to Richter and Akerblom in 1983, following intramuscular or subcutaneous injections of various PEG-modified proteins in Complete Fruend’s Adjuvant^17^. However, immunogenicity directed specifically against PEG was not confirmed until more than a decade later, when multiple groups found APA-induced accelerated blood clearance of subsequent doses of PEG-modified nanoparticles as well as PEGylated proteins, in the absence of adjuvants^18–21^. Recent clinical studies have started assessing induction of APA, and the FDA now requires specific monitoring of APA responses in clinical studies^22^.

The immunogenicity of PEG and the seemingly random induction of APA responses by select PEGylated therapeutics remains poorly understood, and represents an area of active clinical and preclinical research. Current hypotheses point to a number of important factors implicated in the potential APA response including the molecular size of PEG, linker type, number of PEG polymer chains attached, and/or overall immunogenicity of the underlying therapeutic molecule^23–25^. Likewise, it remains poorly understood how APA can specifically bind to the highly flexible and hydrophilic PEG polymer, despite the absence of any fixed conformation to which Ab can directly dock to along the PEG polymer backbone. Lee et al. recently discovered a mechanism by which two different APA clones can bind along the highly flexible PEG polymer backbone^26^.

In this work, we perform structural characterization of a different APA Fab (Clone 6-3)^27^ in complex with PEG antigen using X-ray crystallography to acknowledge a different mechanism by which APA can bind PEG. Our studies reveal a unique, open ring-like structure within this Fab paratope that captures PEG polymer via Van der Waals interactions at the interior and exterior surface of the ring. The ring is formed by virtue of a tryptophan residue residing in the heavy chain complementarity-determining region 3 (HCDR3). We apply single amino acid point mutations at and around this tryptophan residue to confirm the importance of this ring structure for strong binding. Finally, we conduct binding assays to suggest a theoretical molecular mechanism by which this APA clone might bind highly flexible PEG as a polymer antigen.

## Results

### Anti-PEG Fabs bind to PEG as symmetrical dimer complex

**Fig. 1:**
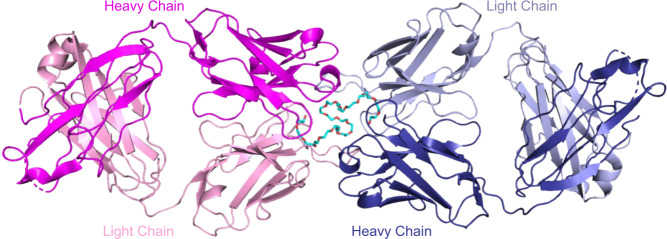
Anti-PEG Fabs form dimer complex with single PEG chain antigen. Overall view of the complex in the asymmetric unit with polyethylene glycol (PEG) polymer antigen (carbon, cyan; oxygen, red) bound between two APA Fabs with color to denote heavy and light chain pairings of each Fab monomer (heavy chain 1, magenta + light chain 1, pink; heavy chain 2, deep blue + light chain 2, light blue).

The complex of our Fab bound to PEG was determined from two different crystal lattices with both revealing virtually identical structures. The first crystal comprised two Fab molecules in the asymmetric unit binding a single coiled PEG molecule between them, with the Fabs and the PEG itself being related by noncrystallographic symmetry (Fig. 1). The second crystal showed a similar arrangement with four Fabs and two PEGs in the asymmetric unit. All the individual Fab–PEG complexes superimposed with RMSD values ranging from 1.1 to 3.9 Å (Supplementary Table S1). When limiting the superpositions to the variable fragment components, the RMSDs dropped to 0.24–0.34 Å, belying flexion about the elbows of the Fabs. The structures superimposed well across every part of the polypeptide chains, including virtually all of the side chain rotamers in the complementarity-determining regions (CDRs), with a few exceptions in cases of absent electron density (Supplementary Fig. S1).

### Open ring structure captures PEG at Fab paratope

**Fig. 2:**
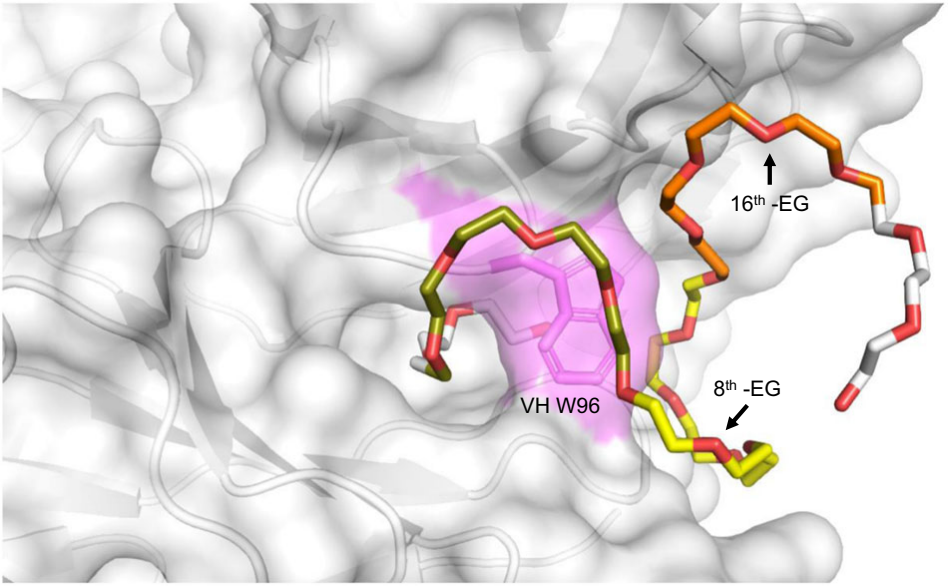
Anti-PEG Fab paratope creates an open ring structure to tightly bind PEG backbone. PEG chain, colored to denote three different semi-circular domains (gold, yellow, and orange) and numbered by each ethylene glycol -EG monomer subunit, is found immobilized within a ring formed by the Fab paratope with Trp96 of HCDR3 (VH W96 labeled magenta) as the key residue sealing off the ring structure. The PEG polymer backbone interacts with both interior and exterior components of the ring-forming paratope folding neatly into three separate semi-circular domains, which creates larger surface area contacts between the Fab paratope and PEG epitope.

Interestingly, the paratope of the APA Fab comprised an open ring that protrudes outward by virtue of Trp96 in CDR3 on the heavy chain (HCDR3) having established a continuous surface feature with Tyr32 and Trp50 on the light chain (Fig. 2). PEG was found linearly immobilized within this ring structure of the Fab paratope, spanning ~3 ethylene glycol (-EG) monomer subunits. The remainder of the crystallized polymer antigen curled back along the exterior surface of the paratope ring structure, wrapping itself neatly into three semicircular domains around the Trp96 residue in HCDR3 and clinging to a broad cleft largely formed by LCDR1 and HCDR1. As such, the immobilized PEG polymer assumed a spiral shape directed outward from the Fab paratope, with solvent water molecules localized near the center of the semicircular domains via alternating hydrogen bonding interactions along the PEG’s inward pointing ether oxygen atoms (Supplementary Fig. S2). Within the antigen–binding interface, the primary interacting residues included Asp95, Trp96, and Gly97 in HCDR3, comprising more of the ring-forming motif. The binding interface also included residues Tyr32 and Ala34 on the light chain, with Tyr32 residue of LCDR1, helping to form the ring structure. Residue Ala34 was in the framework region on the C-terminal end of LCDR1, where it contacted the segment of PEG that passed through the ring. Other light chain residues found at the base of LCDR3, Leu89, and Tyr91, also interacted with PEG in the ring.

By counting the number of monomer repeats of the PEG polymer interacting with the interior and exterior paratope of the Fab, we found the size of the PEG antigen epitope to be ~700 Da, equivalent to 16 monomer subunits (Fig. 2; Supplementary Fig. S3). This epitope size matched well with the prior literature based on ELISA measurements with different sized PEG coatings^28^. We found little evidence of consistent H-bonding interactions between the antibody and ether linkages of the immobilized PEG molecule. It is therefore likely that binding and complex stabilization were largely driven by Van der Waals interactions and burying of surface area, rather than via specific polar interactions. This result is consistent with a broad study of antibody–antigen structures that showed a majority of the most commonly observed contacts were Van der Waals interactions^29^. We found ~500–600 Å^2^ of buried paratope surface area in each Fab–PEG complex.

### Open ring structure is critical for strong binding interactions

**Fig. 3:**
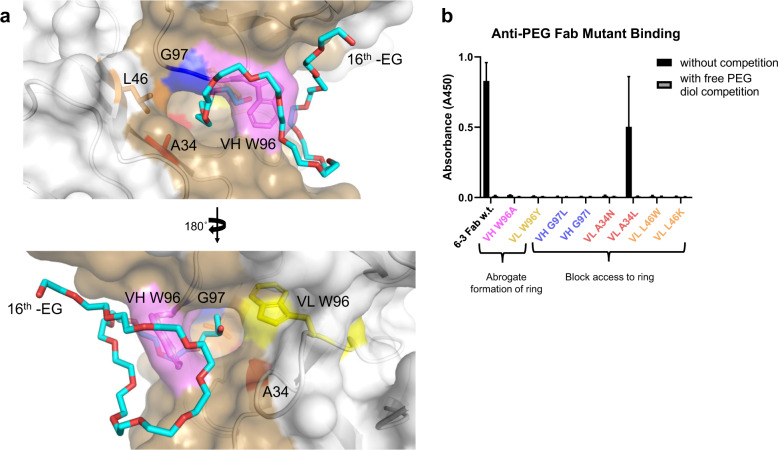
Point mutations to residues of the ring-forming motif abolish antigen binding. **a** Zoomed view of 6-3 Fab paratope, highlighted brown, interacting with truncated PEG chain (carbon, cyan; oxygen, red; numbered by each ethylene glycol -EG monomer subunit) to display specific interactions between Fab and PEG antigen molecules. Amino acid residues contributing to the Fab ring structure, labeled and highlighted various colors, were mutated and tested for binding PEG antigen compared to wildtype Fab. Two categories of mutants were tested: (1) those that might directly eliminate ring structure formation (magenta and yellow), and (2) those that would add larger, more bulky side chain residues to close off the openings of the ring structure (blue, red, and orange). **b** Relative binding of wildtype and mutant Fabs to PEG antigen was compared via direct enzyme-linked immunosorbent assay (ELISA). All mutants able to express displayed reduced binding to PEG antigen with the majority of mutants, seven out of eight, displaying no detectable binding at all. Free PEG diol was added as competition antigen during Fab binding assay to validate specificity. ELISA data is presented from a single, representative technical replicate in which each sample was tested in triplicate. Values are presented as the mean ± standard deviation.

To confirm the importance of this ring structure in the Fab paratope for binding PEG, we performed single amino acid point mutations to residues comprising the internal portion of the paratope’s ring structure and investigated whether these mutant Fabs can still bind PEG. Specifically, we targeted Trp96 and Gly97 of the variable heavy chain and Ala34, Leu46, and Trp96 of the variable light chain (Fig. 3a). We tested two different strategies to abrogate PEG binding: (1) those that might directly eliminate this ring structure (VH W96A and VL W96Y), and (2) those that would add larger, more bulky side chain residues to close off the openings of the ring structure (VH G97L, VH G97I, VL A34N, VL A34L, VL L46W, and VL L46K). We quantified the relative binding of mutants versus wildtype by direct PEG enzyme-linked immunosorbent assay (ELISA) with free PEG competition to ensure antigen specificity^30^. We found no detectable binding for seven of the eight mutant Fabs (Fig. 3b). Mutating the Trp96 residue in HCDR3 to a less bulky hydrophobic side chain (VH W96A) completely eliminated PEG binding, as anticipated since Trp96 serves as the exterior cap closing the ring structure in the paratope. Likewise, most of the mutations adding bulky residues to block the inner opening of the ring structure were successful in eliminating PEG binding as well. The addition of these bulkier amino acid residues along the interior surface of the ring likely either excluded the PEG polymer from fitting inside the open ring or destabilized the open ring structure altogether. VL A34L was the only mutant to retain some degree of PEG binding.

### Binding mechanism involves paratope conformational change

**Fig. 4:**
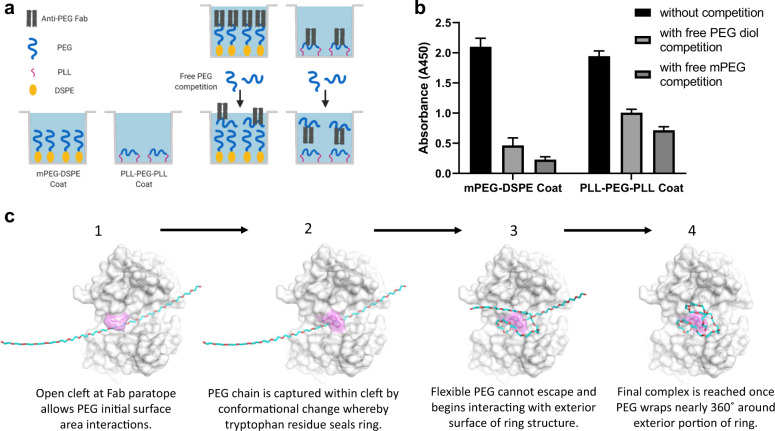
Anti-PEG Fab undergoes conformational change at binding site to create final open ring structure after initial interactions with PEG chain backbone. **a** Cartoon schematic depicting enzyme-linked immunosorbent assays (ELISAs) used to test Fab binding to either single-sided immobilized (mPEG-DSPE coat) or double-sided immobilized (PLL–PEG–PLL coat) PEG. APA Fab could bind mPEG-DSPE coated plates by either allowing free PEG chain to thread through the preformed ring structure of the Fab or undergoing conformational change in the Fab upon initial interaction with PEG backbone to create the final ring structure. By flanking both terminal ends of the PEG chain with large molecular weight PLL polymers and immobilizing these PLL end groups, APA Fab could only bind to the PEG backbone of the triblock co-polymer by undergoing a conformational change at the paratope following initial interaction with PEG chain. **b** Fab binding data for mPEG-DSPE and PLL–PEG–PLL coated ELISAs depicted in part A schematic. In the absence of free PEG competition, Fab binds similarly to both mPEG-DSPE and PLL–PEG–PLL coated plates. Immobilized PEG binding can be competed off similarly with either PEG diol or methoxy-PEG (mPEG) since the Fab is specific to PEG chain backbone. ELISA data are presented from a single, representative technical replicate in which each sample was tested in triplicate. Values are presented as the mean ± standard deviation. **c** Theoretical mechanistic model depicting the step-by-step dynamic processes of anti-PEG Fab binding to flexible PEG antigen. The tryptophan residue in CDR3 of the heavy chain (Trp96, magenta) is essential in sealing the ring structure and stable capture of PEG.

A key question remains in regard to how PEG actually engages with the Fab paratope ring structure to achieve the observed final complex. One possibility is for the PEG polymer to directly thread itself into the open ring, akin to inserting a string through the pinhole of a sewing needle. Alternatively, if the paratope open ring structure is dynamic due to the conformational flexibility of Trp96 in HCDR3, the PEG polymer could initially interact with the underlying open cleft of the paratope, followed by closing of the cleft from rotation of the bulky but flexible Trp96 residue that captures PEG, finally arriving at the structure revealed from the crystals when highly flexible PEG contours around Trp96 and further stabilizes itself on the exterior surface of the ring paratope. To answer this question, we designed a direct PEG ELISA employing two different types of PEG coatings: (1) methoxy-PEG-lipid (mPEG-DSPE) whereby only one end of PEG is modified by a nonpolar lipid side for binding to the ELISA plate, and (2) poly-l-lysine–PEG–poly-l-lysine (PLL–PEG–PLL) triblock co-polymer, where both ends of PEG are modified with 3 kDa molecular weight cationic PLL (Fig. 4a). We found the APA Fab to similarly and specifically bind both PEG coatings (Fig. 4b). Since it is very unlikely that a large molecular weight PLL polymer chain could efficiently thread itself deep into the paratope ring, these results are consistent with the Fab paratope directly engaging ethylene glycol subunits along the PEG backbone as a consequence of conformational fluctuations of the Trp96 residue. In other words, this tryptophan side chain toggles to reveal an interior pocket for PEG to initially associate, followed by sealing of the external cap and capture of PEG, and finally further stabilization by Van der Waals interactions between PEG and the interior surface of the ring structure. The flexible PEG molecule may then sample various conformations, including the aforementioned semi-circular domains. This leads to PEG wrapping itself around the exterior of the tryptophan ring, creating more surface area contacts with various residues of the Fab paratope, and arriving at the final stable bound complex (Fig. 4c).

## Discussion

We have determined here the structural characteristics of an APA in complexation with its polymer antigen. The structure helps to solve some of the mystery shrouding APA over the past several decades by elucidating a mechanism of action against PEGylated therapeutics. The antibody paratope forms a dynamic ring structure that can capture PEG, followed by further stabilization of the binding as PEG wraps itself around the key Trp96 residue of the heavy chain, creating large surface area contacts for significantly strong Van der Waals interactions. Indeed, several other APA clones with available amino acid sequences from the literature possess either the Trp96 residue or a similarly bulky and hydrophobic Tyr96 in the CDR3 of the variable heavy chain (Supplementary Fig. S4). The unique mechanism of binding described here might also explain and further validate some of our previous work where we found APA cross-reactivity to other C–C–O backbone polymers, namely polypropylene glycol (PPG) and poly(1,4-butylene adipate) (PBA), which have similar repeating unit chemical structures and polymer flexibilities as PEG^31^. Either PPG or PBA polymer could likely fit into the Fab paratope’s cleft to engage binding by accumulation of Van der Waals interactions, albeit to a significantly weaker extent than with PEG as antigen.

Recently, Lee et al. determined the structures of two different APA Fab clones in complexation with PEG polymer backbone^26^. Similar to our results, they found a single PEG chain immobilized between two separate Fab molecules in their crystal structures. Using analytical ultracentrifugation with different concentrations of Fab in solution, the group confirmed this Fab dimer formation phenomenon to occur only in the presence of soluble PEG, whereas Fab molecules exist only as monomeric units in the absence of PEG. Their studies provide strong evidence that this dimer complex exists in solution and is not simply an artifact of packing in the crystal lattice. Molecular modeling suggests dimerization by a single IgG molecule would not be possible as the flexible hinge region connecting Fabs to Fc would not provide enough space to simultaneously bind along the same PEG polymer chain. Interestingly, we found completely different APA Fab and PEG conformations when overlaying our 6-3 clone structure with the 3.3 clone structure from Lee et al. (Supplementary Fig. S5). Although both APA Fabs make significant contact with PEG antigen through aromatic side chains of the CDR3 heavy chain (W96 for clone 6-3; Y97 and Y98 for clone 3.3), only our 6-3 clone reveals a unique ring structure for capturing PEG by virtue of its aromatic tryptophan forming an exterior cap within the paratope. Along with considerable differences among the HCDR3 regions, significant divergence is also witnessed between the HCDR2 and LCDR1 domains forming the corresponding Fab paratopes. Furthermore, the PEG molecule presents itself in dramatically different orientations between the different structures. In our structure with the 6-3 Fab clone, we observe PEG in a 3-dimensional spiral shape protruding outward from the Fab paratope. In contrast, the PEG molecule from the 3.3 Fab clone is found in a more 2-dimensional S-shaped orientation. Coupled with prior knowledge from the literature that the 6-3 clone binds to PEG with an apparent affinity 14-fold greater than the 3.3 clone^32^, these contrasting APA structures might suggest that a more 3-dimensional PEG antigen conformation, such as with the 6-3 Fab structure, creates additional surface area interactions in the APA-PEG complex for higher binding affinity. Despite these conformational differences in the structures of different APA clones, the similar amounts of significant buried surface area in the APA–PEG complexes suggest that a minimum buried surface area is needed to confer sufficient binding for an APA to capture PEG.

Taken together, these recent structural insights provide clear mechanisms by which APA can specifically bind seemingly inert PEG polymers. The work also furthers our understanding of humoral immunity generated against such small and highly flexible repeated structures lacking fixed conformations, which drastically differs from typical protein–protein interactions occurring in more classic humoral responses. The structural insights revealed from our work should prove useful beyond just better understanding humoral immunity and will likely guide the future development of next generation stealth polymers that could better prevent and mitigate adverse anti-polymer responses in the body. Similarly, our work may benefit the areas of research in biomedical science harnessing APA for beneficial applications, including development of sensitive immunoassays for detecting PEG-containing compounds^28,33–35^ as well as engineering bispecific APA for enhancing targeted drug delivery^32,36–40^. Through rational, structure-based design of antibody molecules, better PEG-binders could be generated to further improve upon the current successes of these APA immunoassays and bispecific APA targeted drug delivery systems.

## Methods

### Protein production

Separate expression vectors, both under the CAG promoter, were generated by Gibson Assembly for the heavy chain and light chain constructs. Following an albumin signal peptide for protein secretion, the heavy chain construct consisted of the murine anti-PEG backbone variable heavy domain (Clone 6-3)^27^ and human IgG_1_ constant heavy 1 domain (V_H_-C_H1_), and the light chain construct consisted of the murine anti-PEG backbone variable light domain (Clone 6-3)^27^ and human constant kappa light chain domain (V_L_-C_κ_). An 8x polyhistidine tag was added to the C-terminus of the heavy chain plasmid for purification purposes. Plasmids encoding the chimeric heavy and light chains were co-transfected at 1:1 molar ratio into Expi293F mammalian cells using the ExpiFectamine 293 transfection kit (Gibco, Gaithersburg, MD). After ~5 days of recombinant protein expression, cells were pelleted by centrifugation at 10,000 × *g*, and the supernatant containing expressed antibody Fab was harvested and filtered through a 0.2 μm PES filter. Anti-PEG Fab (V_H_-C_H1_/V_L_-C_κ_) was purified from cell culture supernatant via immobilized metal affinity chromatography (IMAC) using Ni-NTA agarose (Qiagen, Germantown, MD) with a gravity column. Purified protein was simultaneously concentrated (>1 mg per mL) and buffer exchanged into either PBS (binding assays) or Tris buffer (crystallography) using ultrafiltration (MWCO 30 K, Amicon Ultra). Fab concentration was determined by spectrophotometry measurements using calculated protein extinction coefficients (A280 NanoDrop^TM^ One/One^C^) and confirmed by BCA protein assay (Pierce, Rockford, IL). Size and purity of Fab (Supplementary Fig. S6) were assessed by sodium dodecyl sulfate polyacrylamide gel electrophoresis (SDS-PAGE), and protein bands were detected with Coomassie stain (Imperial protein stain, Thermo Scientific, Waltham, MA).

### Crystallography

Prior to crystallography procedures, purified Fab was buffer exchanged into 20 mM Tris HCl pH 7.5, 50 mM NaCl and brought to a final sample concentration of 10.6 mg per mL. The unliganded Fab was tested for crystallization against commercially available sparse matrix screens. SDS format sitting drop plates were used with automation (Douglas Instruments Ltd). Drops composed of 0.15 μL protein with 0.15 μL reservoir were set up against 30 μL reagent reservoirs. Diffraction-quality crystals were obtained in two reagent conditions: (1) 20% PEG 4 K MW, 100 mM sodium citrate/citric acid pH 5.5, 10% isopropanol, and (2) 25% PEG 1500 MW, 100 mM MMT buffer pH 6.5. The crystals were briefly soaked in reservoir supplemented with 15% ethylene glycol prior to cryocooling in liquid nitrogen, followed by data collection. Diffraction data were collected at SER-CAT, Advanced Photon Source, using an incident beam of 1 Å in wavelength. Data were reduced in HKL-2000^41^. The structures were phased by molecular replacement in PHENIX^42^ using the paired heavy and light chains of 1AJ4 and 6ANA, respectively, as search models and separated into variable fragment and constant domain components^43,44^. The liganded PEG molecules were built into positive difference density as part of manual rebuilding in Coot and refinement in PHENIX^42,45^. Coordinates and structure factors have been deposited in the Worldwide Protein Data Bank (wwPDB) with accession numbers 6VL9 and 6VL8. Analysis was facilitated by PDBePISA^46^. Figures were generated in PyMol (The PyMOL Molecular Graphics System, Version 1.2r3pre, Schrödinger, LLC).

### Site-directed mutagenesis

To impart single amino acid mutations in the variable domains of the parental wildtype expression plasmids, we performed site-directed mutagenesis with Gibson Assembly cloning. First, we designed pairs of overlapping mutagenic primer sets and amplified mutant gene fragments by PCR using the parental wildtype expression plasmids as template. Following PCR, the amplicons were cleaned up by Monarch^®^ PCR/DNA clean up kit (New England Biolabs, Ipswich, MA). Parental wildtype heavy and light chain expression plasmids were linearized by double restriction enzyme digest at sites flanking the gene insert region using Acc65I and XhoI. The overlapping mutagenic PCR products and linearized backbone vector were joined together with Gibson Assembly Master Mix (New England Biolabs, Ipswich, MA). The new mutant plasmids were transformed into One Shot TOP10 chemically competent cells (Thermo Scientific, Waltham, MA) and plated on LB-carbenicillin plates for colony screening. Several colonies from each plate were screened by Sanger sequencing (Genewiz, South Plainfield, NJ) to confirm appropriate mutations in the variable domains.

### ELISA to characterize PEG binding

Direct PEG enzyme-linked immunosorbent assay (ELISA) was used to characterize and compare the relative binding between wildtype and mutant Fabs^30^. Methoxy-PEG-lipid (mPEG-DSPE with 5k MW PEG, Nanocs) was coated to the bottom of clear, non-treated, half-area 96-well plates (Corning Costar 3695) at a final dilution of 50 μg per mL in PBS by overnight incubation at 4 °C. Unbound mPEG-DSPE was washed away by 5× PBS washes of the wells. Nonspecific binding was blocked by incubating wells with 5% weight per volume nonfat milk in PBS for 1–2 h at room temperature. Purified wildtype and mutant anti-PEG antibody (APA) Fabs were diluted to 500 nM final concentration in 1% weight per volume milk-PBS and incubated for 1 h at room temperature. Excess free PEG diol (8k MW PEG, Sigma-Aldrich) was added to certain wells during APA Fab incubation to demonstrate PEG backbone specificity and compete off binding to plate-bound mPEG-DSPE. After primary antibody incubation, plate wells were washed 5× with PBS to remove unbound APA Fab. Bound APA Fabs were detected using goat anti-human kappa light chain HRP conjugated secondary antibody (Sigma-Aldrich, cat no. A7164) at 1:1000 dilution in 1% weight per volume milk-PBS for 1 h incubation at room temperature. Following 5× PBS washes to remove unbound secondary antibody, 1-step Ultra TMB-ELISA substrate solution (Thermo Scientific, Waltham, MA) was added for up to 30 min to detect HRP activity. The enzymatic reaction was quenched by adding equal volume of 2 N sulfuric acid, and the color development was immediately determined by taking absorbance measurements at 450 nm (signal) and 570 nm (background) wavelengths using a SpectraMax M2 microplate reader (Molecular Devices, San Jose, CA). Negative control wells, including PEG-coated, blocked wells without primary APA Fab incubation and uncoated, blocked wells with primary APA Fab incubation, both revealed only negligible signal in the assays.

Direct PEG ELISA was also used to characterize and compare relative binding of wildtype APA Fab to two different immobilized PEG formats: (1) mPEG-DSPE as before, whereby only one end of the PEG chain is modified by non-polar lipid for direct binding to the well bottoms of a 96-well plate, and (2) poly-l-lysine–PEG–poly-l-lysine (PLL–PEG–PLL) triblock co-polymer, whereby both ends of PEG are modified with high MW cationic PLL for direct binding of both terminal ends to the well bottoms of a 96-well plate. Either mPEG-DSPE (5k MW PEG, Nanocs) at 50 μg per mL final concentration in PBS or PLL-PEG-PLL (3k MW PLL; 5k MW PEG, Nanosoft Polymers) at 100 μg per mL final concentration in PBS was coated to the bottom of clear, non-treated, half-area 96-well plates (Corning Costar 3695) by overnight incubation at 4 °C. Unbound PEG coatings were washed away by 5× PBS washes of the wells. Nonspecific binding was blocked by incubating wells with 5% weight per volume bovine serum albumin (BSA) in PBS for 1–2 h at room temperature. Purified wildtype APA Fab was diluted to 500 nM final concentration in 1% weight per volume BSA–PBS and incubated for 1 h at room temperature. Excess free PEG diol (8k MW PEG, Sigma-Aldrich) or free mPEG-Amine (5k MW PEG, JenKem Technology USA) was added to certain wells during APA Fab incubation to demonstrate PEG backbone specificity and compete off binding to plate-bound PEGs. After primary antibody incubation, plate wells were washed 5× with PBS to remove unbound APA Fab. Bound APA Fabs were detected using goat anti-human kappa light chain HRP conjugated secondary antibody (Sigma-Aldrich, cat no. A7164) at 1:1000 dilution in 1% weight per volume BSA-PBS for 1 h incubation at room temperature. Following 5× PBS washes to remove unbound secondary antibody, 1-step Ultra TMB-ELISA substrate solution (Thermo Scientific, Waltham, MA) was added for up to 30 min to detect HRP activity. The enzymatic reaction was quenched by adding equal volume of 2 N sulfuric acid, and the color development was immediately determined by taking absorbance measurements at 450 nm (signal) and 570 nm (background) wavelengths using a SpectraMax M2 microplate reader (Molecular Devices, San Jose, CA). Negative control wells, including PEG-coated, blocked wells without primary APA Fab incubation and uncoated, blocked wells with primary APA Fab incubation, both revealed only negligible signal in the assays.

### Variable domain amino acid sequence alignment

The variable domains, including both V_H_ and V_L_, from various APA clones found in the literature were compared by multiple amino acid sequence alignment using Clustal Omega. Residues were numbered according to Kabat numbering, and CDRs were identified using the abYsis online resource. APA clones 6-3, 3-3, E11, PEG.2 6A9, and 157G29D1 are specific to the PEG repeating monomer backbone as a binding epitope, while APA clone 15-2 is specific to the terminal methoxy-PEG (mPEG) end group as a binding epitope.

### Bio-layer interferometry to measure binding kinetics

An Octet RED384 instrument (ForteBio, Fremont, CA) was used for BLI to determine binding kinetics and affinity between APA 6-3 Fab and PEG antigen (Supplementary Fig. S7). mPEG-Amine (3k MW PEG, JenKem Technology USA) was immobilized onto the surface of amine reactive 2nd generation (AR2G) biosensor probes (ForteBio) via covalent amide bond formation through standard EDC-reaction chemistry. Briefly, AR2G probes were regenerated for at least 10 min in water prior to PEGylation reaction overnight at room temperature with 10 mM mPEG-Amine and 100 mM EDC in 50 mM borate buffer at pH 7.8. The PEGylation reaction was quenched the following morning by transferring the probes into a 50 mM borate buffer solution containing 200 mM glycine and 200 mM EDC at pH 7.8. Following the 2 h quench at room temperature, probes were transferred into BLI assay buffer to wash off excess PEG, EDC, and glycine prior to measurements on the Octet. BLI assay buffer was used for the washing of probes as well as in the baseline, association, and dissociation steps of the binding kinetic assay. BLI assay buffer consisted of standard PBS supplemented with 0.02% weight per volume CHAPS and 0.1% weight per volume BSA. APA Fab stocks were serially diluted into BLI assay buffer and used as the analyte in the association step to bind probes with immobilized PEG. Background signal was subtracted from all sample probes using a reference probe that was loaded with PEG antigen but did not receive APA Fab for binding. Other control probes were tested to confirm presence of PEG on the biosensor surface as well as demonstrate insignificant non-specific binding of APA Fab to non-PEGylated biosensors. Reference subtracted sample data from serially diluted APA Fab probes was fit globally using a 1:1 binding model to calculate the kinetic parameters of association rate constant (*k*_on_), dissociation rate constant (*k*_off_), and affinity constant (*K*_D_) for the biomacromolecular interaction.

## Supplementary information

Supplementary Information

## Data Availability

The datasets generated during and/or analyzed during the current study are publicly available from the Worldwide Protein Data Bank (wwPDB) with the accession numbers 6VL9 and 6VL8. All other datasets generated during and/or analyzed during the current study are available from the corresponding author on reasonable request.
