# Supplementary material for: Structure of an anti-PEG antibody reveals an open ring that captures highly flexible PEG polymers
Source: Commun Chem. 2020 Sep 8;3:124. doi: 10.1038/s42004-020-00369-y (PMC9814744; doi:10.1038/s42004-020-00369-y)
Supplement: Supplementary file 1 — Supplementary Information [file 42004_2020_369_MOESM1_ESM.pdf]

Structure of an anti-PEG antibody reveals an open ring that captures highly flexible PEG polymers

Huckaby et al.

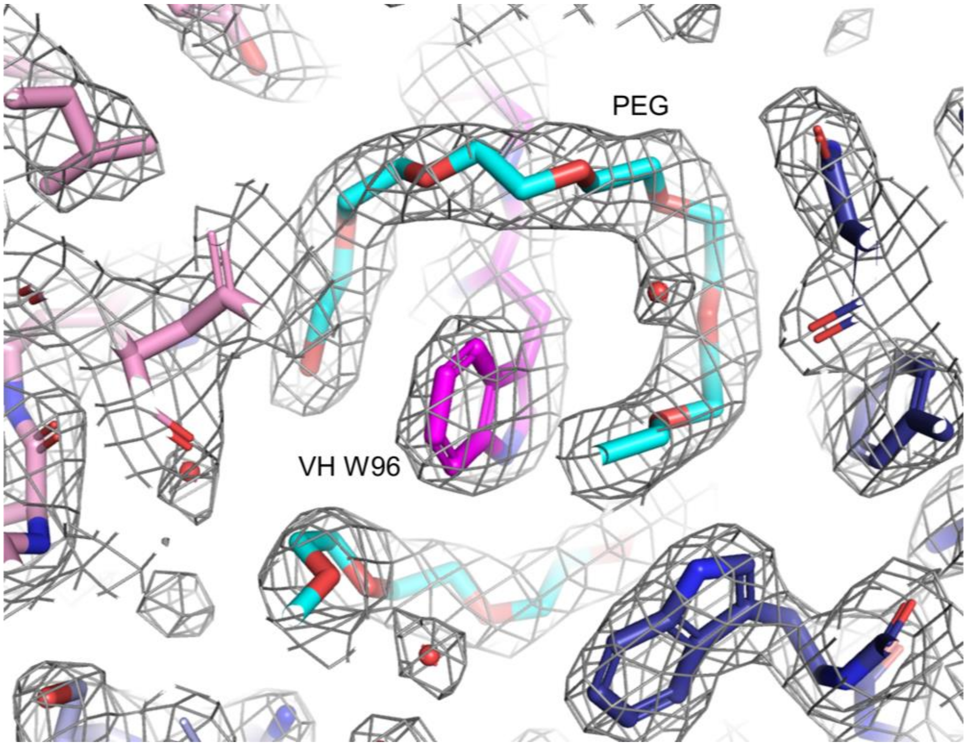

Supplementary Figure 1: Representative electron density for the Fab-PEG interface in the crystal structure

Image showing the 2Fo-Fc electron density map for the crystal structure contoured at a 1sigma level to show the quality of the data in the vicinity of the PEG molecule and the correspondingly bound Fab.

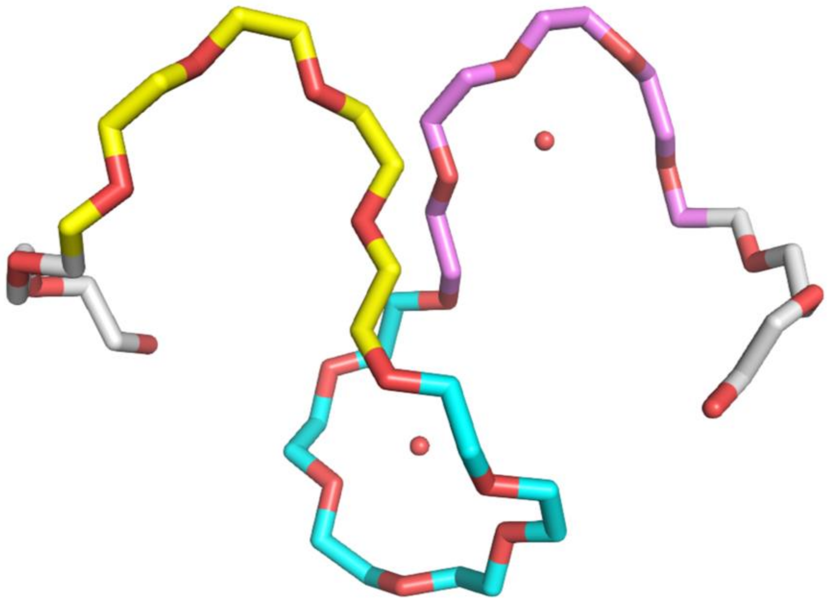

Supplementary Figure 2: PEG polymer antigen colored by semi-circular domain with relevant solvent molecules added

Water solvent molecules make alternating hydrogen bonds to ether oxygen atoms of the PEG polymer chain, contributing to the various semi-circular domains (colored yellow, cyan, and magenta) and overall spiral shape of the PEG molecule while in complex with the anti-PEG Fab.

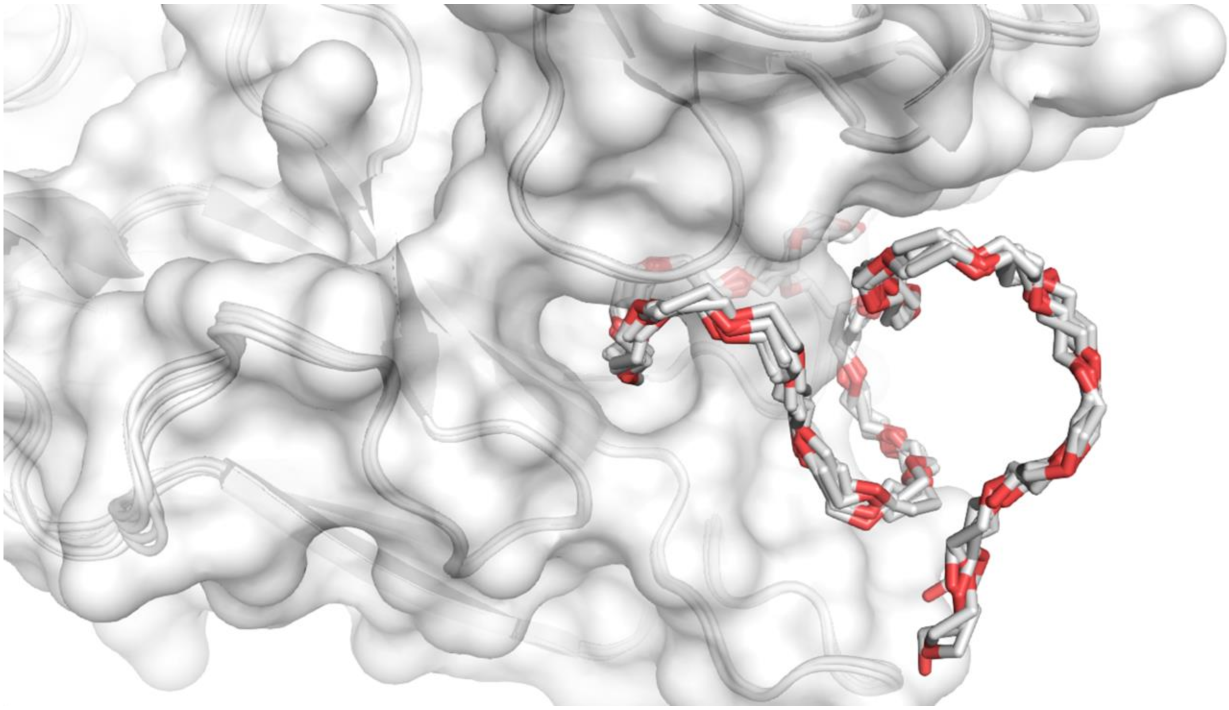

Supplementary Figure 3: Superposition of all antibody-antigen structure complexes

Superimposing all complexes from both of the crystal structures reveals strong alignment for protein atoms of the Fab molecules as well as atoms along the PEG antigens, but alignment begins to diverge near the terminal ends of the polymer chains. This divergent behavior is likely a result of inherent PEG polymer chain flexibility distal to the antigen-binding interface region of the complex.

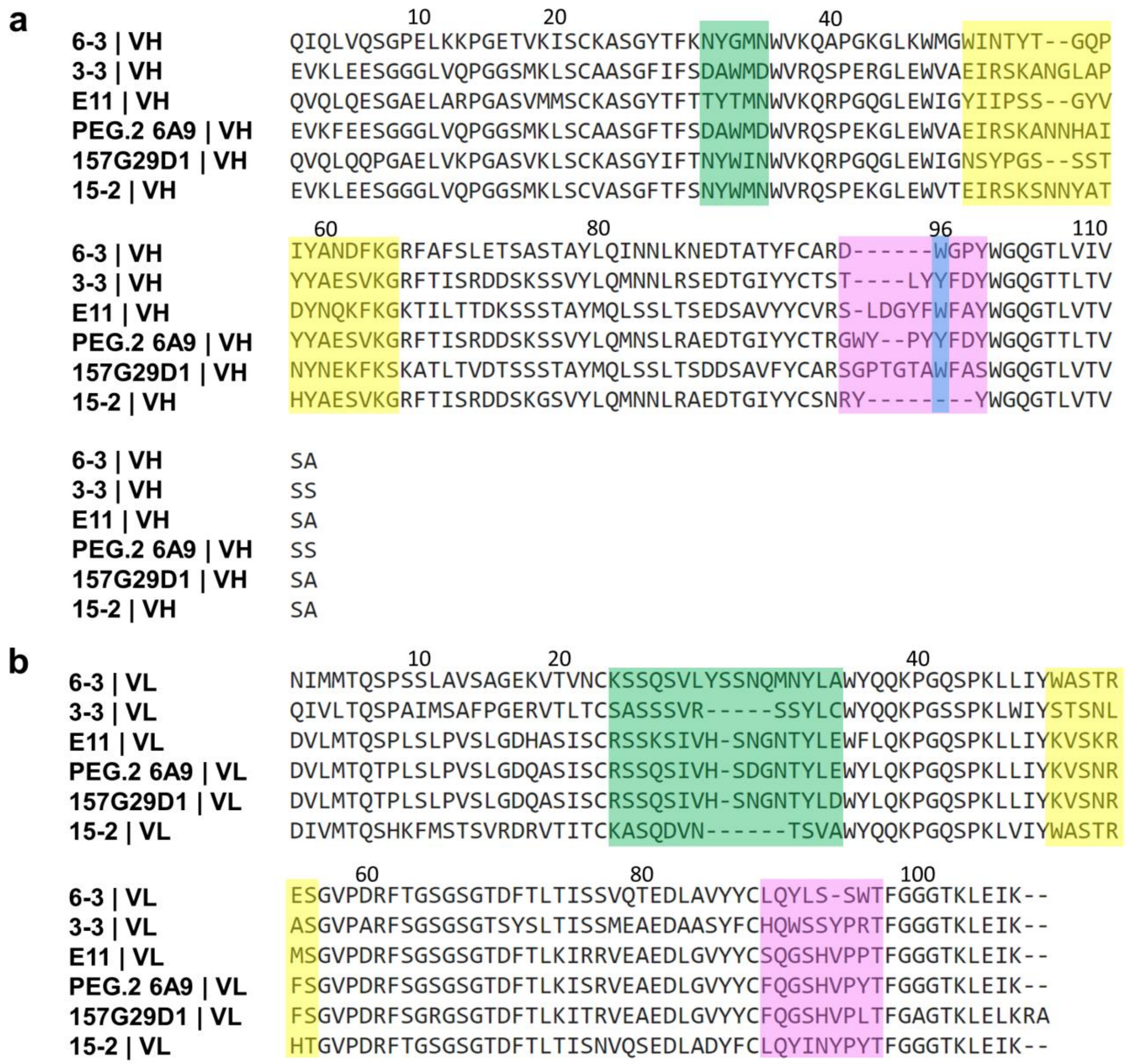

Supplementary Figure 4: Variable domain amino acid sequence alignments for various APA clones

a Variable heavy (VH) domain amino acid sequence alignments for six different APA clones (6-3, 3-3, E11, PEG.2 6A9, 157G29D1, and 15-2) with Kabat numbering and CDRs highlighted (CDR1, green; CDR2, yellow; CDR3, pink). The 96th residue is labeled and highlighted blue to show conservation of the ring-forming tryptophan (W) or

similarly bulky and hydrophobic tyrosine (Y) residue across other APA clones in the VH domain.

b Variable light (VL) domain amino acid sequence alignments for six different APA clones (6-3, 3-3, E11, PEG.2 6A9, 157G29D1, and 15-2) with Kabat numbering and CDRs highlighted (CDR1, green; CDR2, yellow; CDR3, pink).

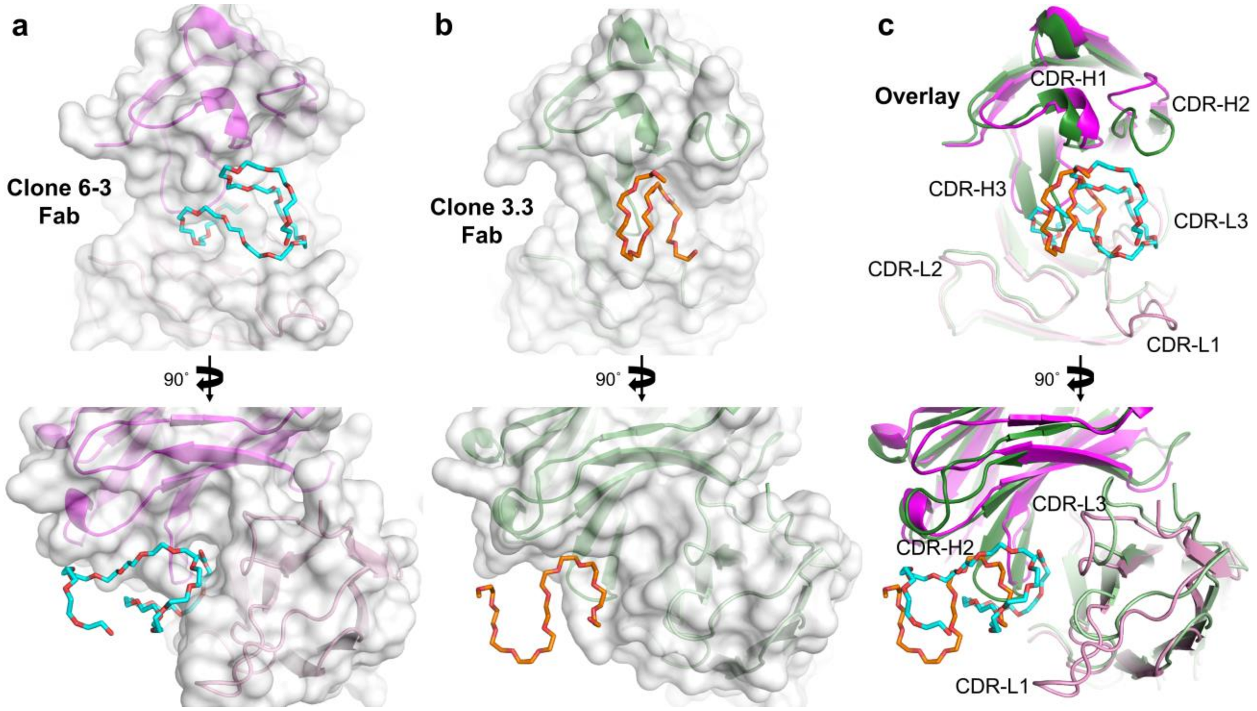

Supplementary Figure 5: Overlay of two different APA Fab clone structures reveals different binding mechanisms to PEG backbone

a Ribbon (magenta) and surface model representations of clone 6-3 Fab structure in complexation with PEG backbone antigen (carbon, cyan; oxygen, red).

b Ribbon (green) and surface model representations of clone 3.3 Fab structure in complexation with PEG backbone antigen (carbon, orange; oxygen, red).

c Overlay of clone 6-3 Fab (magenta) and clone 3.3 Fab (green) structures represented as ribbon models in complex with PEG backbone antigens. Complementarity-determining regions (CDRs) in the variable domains at the antigen-binding interface are labeled to show structural differences in the hypervariable regions leading to different PEG binding mechanisms.

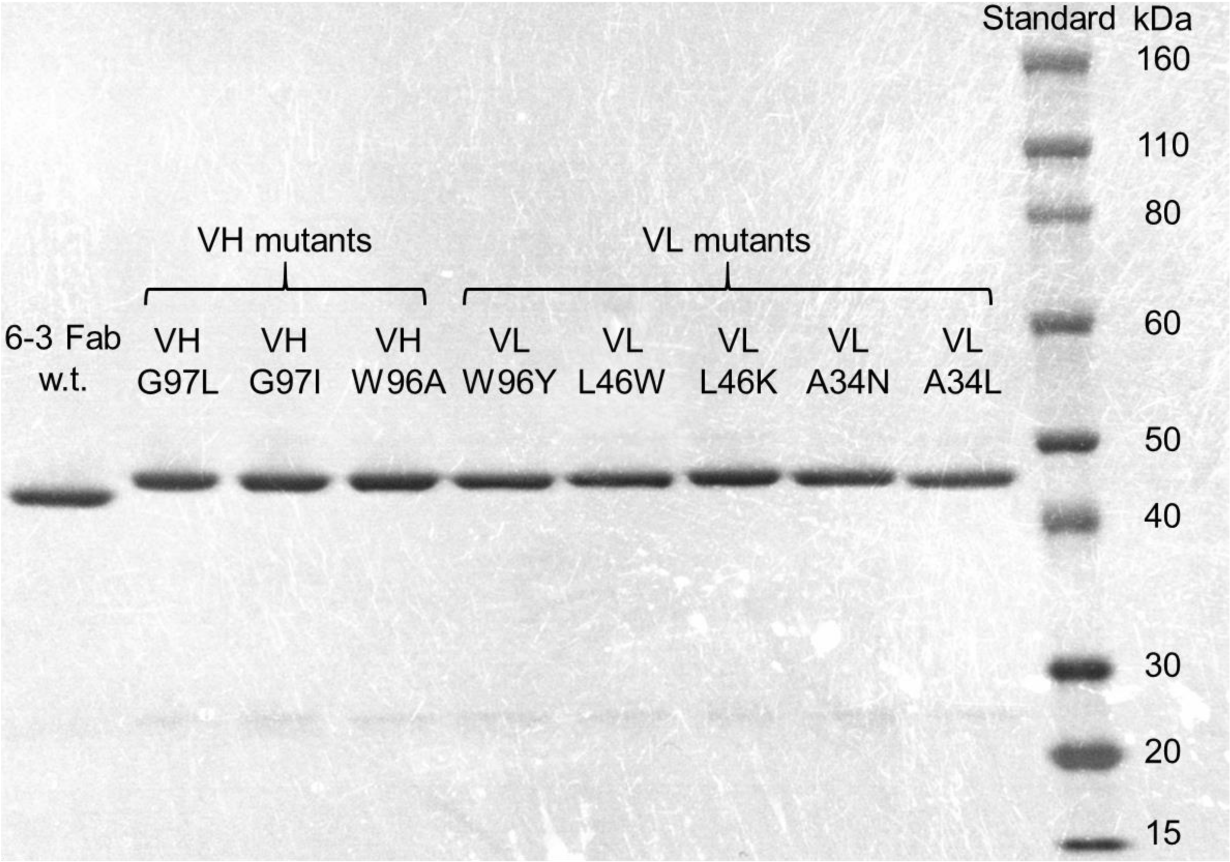

Supplementary Figure 6: Size and purity of wild type (w.t.) and mutant anti-PEG Fabs

SDS-PAGE stained with Coomassie revealed high purity (single stained protein band per sample well) and similar molecular sizes (~ 50 kDa) across all APA Fabs expressed, including wild type (w.t.) and mutant samples.

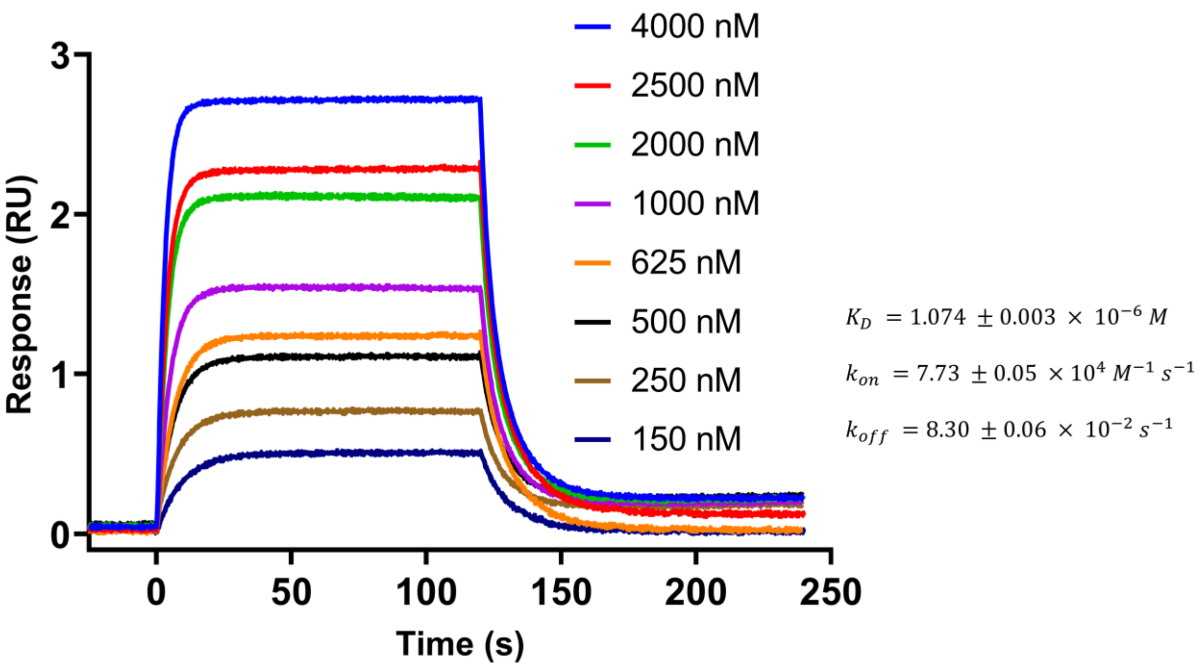

Supplementary Figure 7: Anti-PEG Fab binding kinetics and affinity to PEG polymer backbone antigen

Bio-layer interferometry (BLI) was used to measure the binding affinity constant (K_D), association rate constant (k_{on}), and dissociation rate constant (k_{off}) between APA Fab and PEG antigen. Binding to immobilized PEG was measured at various APA Fab concentrations above and below the calculated binding affinity constant.

Supplementary Table 1: Crystallographic data collection and model refinement statistics

	6VL9	6VL8
Space group	P 21 21 2	P 1 21 1
Unit cell parameters *a*, *b*, *c* (Å)	90.711, 169.788, 69.394	112.108, 87.791, 116.074
Unit cell parameters α, β, γ (°)	90.00, 90.00, 90.00	90.00, 113.66, 90.00
Resolution (Å)	43.819-2.634 (2.700-2.634)[1]	41.925-2.423 (2.484-2.423)
No. of reflections collected	189233	477285
No. of unique reflections	31825	78203
CC1/2	0.973 (0.863)	0.981 (0.916)
CC*	0.993 (0.963)	0.995 (0.978)
Mean I/σI	11.9 (3.08)	17.07 (2.42)
Rpim	0.86 (0.238)	0.075 (0.262)
Completeness (%)	98.0 (100.0)	99.8 (98.6)
Redundancy	5.9 (6.4)	6.1 (4.5)
R_{work}/ R_{free} (%)	20.95/26.28 (27.33/33.31)	20.76/25.67 (28.57/31.00)
Number of protein atoms (non-hydrogen)	6545 (2 Fabs in the AU)	13106 (4 Fabs in the AU)
Number of ligand atoms	59	144 unique (288 total)
Number of solvent atoms (non-hydrogen)	78	168
Average B value for protein atoms (Å^2)	38.10	44.78
Average B value for ligand atoms (Å^2)	36.98	41.34
Average B value for solvent atoms (Å^2)	31.81	37.63
RMSD bond lengths (Å)	0.002	0.003
RMSD bond angles (º)	0.542	0.583
Clashscore	3.77	3.85
Ramachandran favored (%)	96.20	96.33
Ramachandran outliers (%)	0.36	0.36
[1]Values in parentheses correspond to the highest resolution shell.		
